# Nutrient and herbivore alterations cause uncoupled changes in producer diversity, biomass and ecosystem function, but not in overall multifunctionality

**DOI:** 10.1038/s41598-017-02764-3

**Published:** 2017-06-01

**Authors:** J. Alberti, J. Cebrian, F. Alvarez, M. Escapa, K. S. Esquius, E. Fanjul, E. L. Sparks, B. Mortazavi, O. Iribarne

**Affiliations:** 1 Instituto de Investigaciones Marinas y Costeras (IIMyC), UNMdP – CONICET, Mar del Plata, Argentina; 2 Dauphin Island Sea Lab, Dauphin Island, AL 36528 United States; 3 University of South Alabama Department of Marine Sciences, Mobile, AL 36688 United States; 4 Laboratorio Cuenca del Salado, Instituto de Limnología “Dr. Raúl A. Ringuelet” (ILPLA) (CONICET – UNLP), La Plata, Argentina; 5 Laboratorio de Limnología, Departamento de Biología, Universidad Nacional de Mar del Plata, Mar del Plata, Argentina; 6 Mississippi State University Coastal Research and Extension, Biloxi, MS 39532 United States; 7 Mississippi-Alabama Sea Grant Consortium, Ocean Springs, MS 39564 United States

## Abstract

Altered nutrient cycles and consumer populations are among the top anthropogenic influences on ecosystems. However, studies on the simultaneous impacts of human-driven environmental alterations on ecosystem functions, and the overall change in system multifunctionality are scarce. We used estuarine tidal flats to study the effects of changes in herbivore density and nutrient availability on benthic microalgae (diversity, abundance and biomass) and ecosystem functions (N_2_-fixation, denitrification, extracellular polymeric substances -EPS- as a proxy for sediment cohesiveness, sediment water content as a proxy of water retention capacity and sediment organic matter). We found consistent strong impacts of modified herbivory and weak effects of increased nutrient availability on the abundance, biomass and diversity of benthic microalgae. However, the effects on specific ecosystem functions were disparate. Some functions were independently affected by nutrient addition (N_2_-fixation), modified herbivory (sediment organic matter and water content), or their interaction (denitrification), while others were not affected (EPS). Overall system multifunction remained invariant despite changes in specific functions. This study reveals that anthropogenic pressures can induce decoupled effects between community structure and specific ecosystem functions. Our results highlight the need to address several ecosystem functions simultaneously for better ecosystem characterization and management.

## Introduction

There is an increasing number of studies documenting large-scale human impacts on ecosystems, including species losses and altered trophic food webs, as well as nutrient and carbon cycles^1–4^. As a consequence of human activities (e.g., eutrophication, altered consumer populations), pervasive effects on ecosystem functions and services have also been reported^5, 6^. A pressing challenge for environmental managers is to maximize one ecosystem function or service while not hindering others^2, 7^. Thus, there is a need to evaluate several functions or services at once for a complete understanding of how disturbances are altering ecosystems^8, 9^.

Humans have greatly modified the global N cycle, leading for instance to high loads of atmospheric N-deposition^10^. Similarly, human activities have induced dramatic changes in both aquatic and terrestrial ecosystems through altering consumer populations^6^. These two top-down (i.e., consumers) and bottom-up processes (i.e., nutrients) generally influence community structure with opposing effects on the biomass^11^ and diversity of primary producers^12, 13^. Along with impacts on community structure, nutrients and consumers can also affect ecosystem functions and services that support human wellbeing^6, 14^. For example, increased nitrogen availability in terrestrial systems weakens stability in primary production, increases acidity of soils, alters nitrification/denitrification rates and leads to losses of other soil nutrients, plant species richness, and their associated fauna^14, 15^. In aquatic systems, increased nutrient delivery affects nitrogen and carbon cycling, nutrient retention, water quality, and fisheries^16, 17^. These changes can also lead to extensive marsh habitat loss, with persistent changes in soil conditions and dramatic negative impacts for associated biota^18^. Conversely, consumers have been reported to regulate the frequency of fires and soil nutrients in terrestrial systems^6^, and affect nutrient and water retention, as well as decomposition and primary production in aquatic systems^19, 20^.

Research to date suggests that bottom-up or top-down processes may have disparate effects on ecosystem functioning^21, 22^. However, few studies explicitly considered how top-down and bottom-up factors influence several functions simultaneously and examined overall effects on system functionality, particularly in marine systems. Within this context, we evaluated the separate and interactive effects of bottom-up (nutrient enrichment) and top-down (herbivory) anthropogenic impacts on the structure and function of intertidal estuarine sediment flats. We focused on this system because estuaries worldwide are suffering from increased nutrient inputs^17^ and altered consumer populations^23^. In particular, we evaluated (1) how nutrient addition and herbivory affect the structure of benthic microalgae in tidal flats and their associated functions, (2) if these impacts have consistent effects on different ecosystem properties and functions, and (3) their overall structural and functional change. Based on the above information we expected consistent structural changes (positive effects of nutrients and negative effects of herbivory), disparate functional changes, and changes in the overall functioning in the different scenarios.

Benthic microalgae are the dominant primary producers in tidal flats. Their abundance, diversity and biomass is controlled to a variable extent by top-down and bottom-up factors^24, 25^. Among the many potential ecosystem functions directly or indirectly associated with benthic microalgae, we evaluated N_2_-fixation, denitrification, extracellular polymeric substances (EPS), sediment organic matter and water content. Indeed, soil moisture, organic matter (and closely related variables) and components of the N cycle were commonly used in papers to address multifunctionality^8, 9, 26, 27^. Diatoms, a prominent component of benthic microalgal assemblages, are well known for their production of EPS^28, 29^, which can play key roles in these systems because they bind sediments and reduce erosion as well as chelate toxic metals and other contaminants^28^. Cyanobacteria, another relevant component of benthic microalgal assemblages, commonly use atmospheric N_2_ as a source of N^30^. Although benthic microalgae do not directly perform denitrifiaction, in high densities benthic microalgae can outcompete nitrifying bacteria, decreasing coupled nitrification-denitrification rates^31^. Additionally, higher abundance of benthic microalgae can also be associated with higher sediment organic matter, particularly in the upper layer^32^, and thus also to sediment water content, given that sediment water content usually follows similar patterns as organic matter^33, 34^.

## Results

### Structural and functional changes

**Figure 1:**
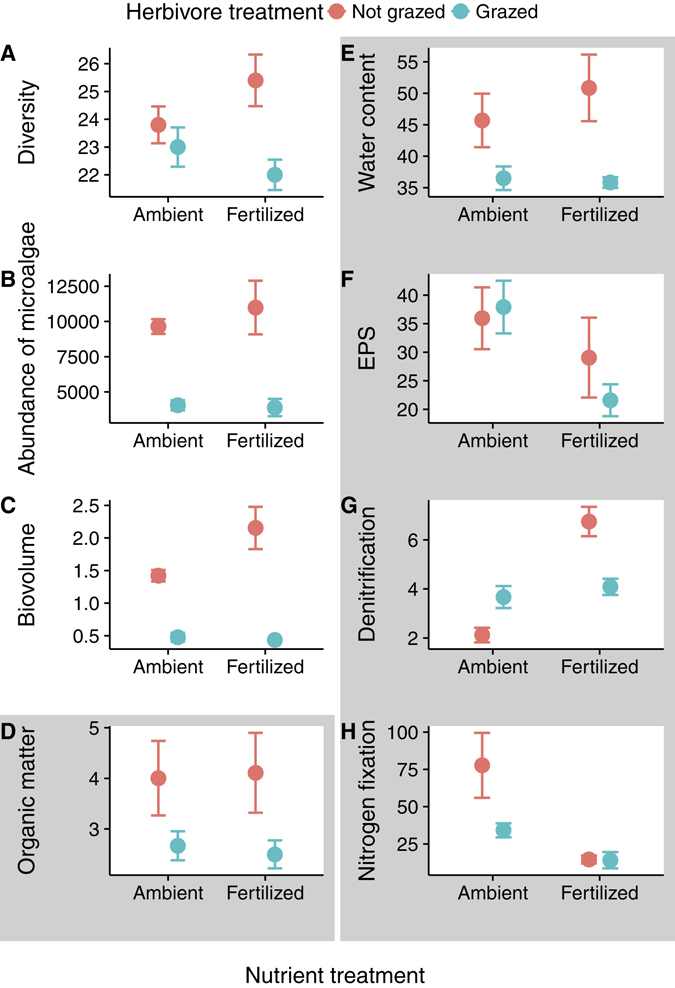
Effect of increased nutrients and altered consumer populations on the (**A**) diversity, (**B**) abundance (individuals mm^−2^) and (**C**) biovolume (mm^3^ cm^−2^; an estimate of biomass) of benthic microalgae, and ecosystem functions (**D**): organic matter (%), (**E**): water content (%), (**F**): EPS (μg glucose cm^−2^), (**G**): denitrification (μmol N_2_ m^−2^ h^−1^), and (**H**): Nitrogen fixation (μmol N_2_ m^−2^ h^−1^). Mean ± SE are represented. Statistical results are given on Supplementary Table 1. Here and in the next figures, the grayed background is used to highlight results of ecosystem functions.

Our results showed that both nutrient addition and herbivory affected the structure of benthic microalgal communities in intertidal flats. These effects were consistent; herbivorous snail density strongly affected the abundance, diversity and biomass of benthic microalgae. However, nutrient addition weakly affected these structural variables (Fig. 1, Supplementary Table 1). In general, snails largely reduced microalgal biomass (75%), abundance (58% and 65% in ambient and fertilized treatments, respectively) and diversity (3% and 13% in ambient and fertilized treatments, respectively). Nutrient addition increased microalgal abundance and diversity, but these changes occurred only in a subset of grazing treatments and were small in magnitude (14% higher abundance in fertilized exclosures and 18% higher diversity in fertilized controls relative to the non-fertilized treatment). Nutrient addition and herbivory also affected ecosystem functions but, in clear contrast to what is observed for benthic microalgal community structure because the effects were not consistent among functions. Sediment organic matter and water content followed a similar response to benthic microalgal structure, with snails largely reducing those contents (36% and 25% reductions respectively) and nutrient addition having no effect (Fig. 1, Supplementary Table 1). Despite the large snail-induced reduction in benthic microalgal abundance, they did not reduce sediment EPS concentration. Nutrient addition had little effect on sediment EPS concentration, with only a significant reduction (43%) observed for cage controls (Fig. 1, Supplementary Table 1). Nutrient addition strongly reduced nitrogen fixation (72%), while it was not affected by snails, in spite of the large reduction in benthic microalgal abundance and altered community composition caused by them (Fig. 1, Supplementary Table 1). Snails increased denitrification under non-fertilized conditions (65% higher), and decreased denitrification under fertilized conditions (42% lower). Moreover, nutrient addition increased denitrification in the absence of grazers (218% higher).

### Relationship between community composition and functioning

**Figure 2:**
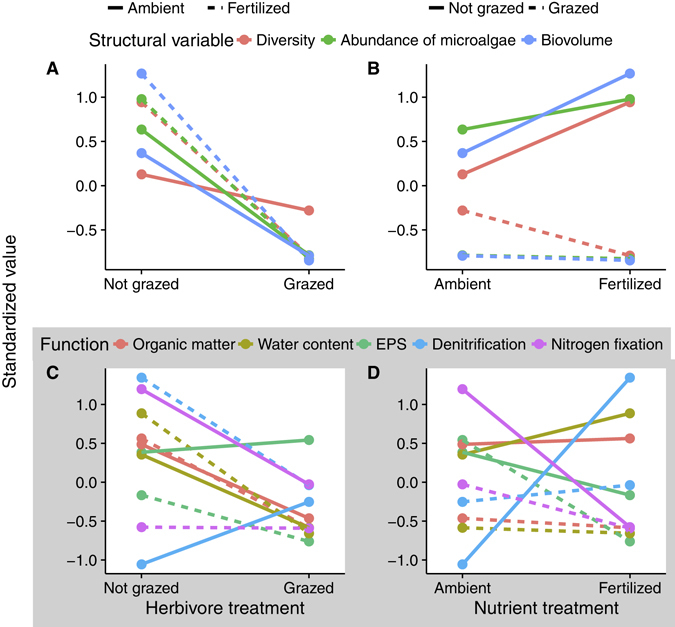
Effect of increased nutrients and altered consumer populations on the standardized diversity, abundance and biomass of benthic microalgae (**A**,**B**) and five standardized ecosystem functions (**C**,**D**). Each color represents the mean response to the treatments.

**Figure 3:**
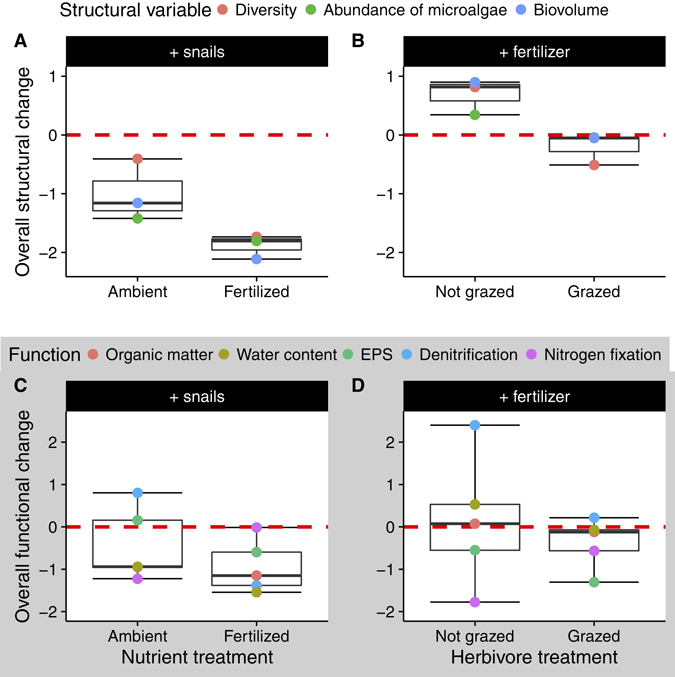
Overall structural (**A**,**B**) and functional (**C**,**D**) change in the different treatments. The dashed red line denotes the “no-change” level.

The effect of each treatment was mostly consistent across structural variables (Fig. 2A,B), while the effect on a given function varied with the treatment or function considered (Fig. 2C,D). The addition of consumers (either with ambient or increased nutrients) or the addition of fertilizer (without grazers) led to marginally significant overall changes in structural variables (respectively, *t*_*2*_ = −3.28, −16.26, 3.97; *p* = 0.082, 0.004, 0.058; Fig. 3A,B), while the addition of fertilizer in grazed plots did not lead to change(*t*_*2*_ = −1.30; *p* = 0.32; Fig. 3B). On the contrary, the addition of fertilizer (either with or without grazers) or the addition of grazers (without fertilization) did not promote an overall functional change (respectively, *t*_*4*_ = −1.39, 0.20, −1.11; *p* = 0.24, 0.85, 0.33; Fig. 3C,D). The only exception was the addition of grazers with fertilization, which induced a negative overall functional change (*t*_*4*_ = −3.33; *p* = 0.03; Fig. 3C). Overall, changes in functioning induced by the nutrients and/or herbivores were not consistent across functions.

## Discussion

Our results show that snail grazing, and nutrients to a lesser extent, consistently regulate all measured variables associated with the microphytobenthos. Interestingly, their impact was not as consistent across different ecosystem functions. Sediment organic matter and water content were affected by snail grazing, N_2_-fixation was strongly and EPS weakly affected by nutrients, and denitrification was affected by the interaction of both factors. In fact, neither snail grazing nor increased nutrients had consistent positive or negative effects on the overall ecosystem functioning. These results imply that prevalent anthropogenic impacts of altered consumer populations and nutrient inputs, two of the most widespread anthropogenic impacts, have decoupled effects between community structure and ecosystem multifunctionality. Our results also suggest that ecosystem functions may not necessarily respond to changes in the biomass, abundance or diversity of primary producers, and that anthropogenic drivers have different impacts on different ecosystem functions.

The impact of grazers and nutrients on benthic micro- and macroalgae has been intensively studied in a variety of aquatic systems (e.g., sandy shallow subtidal^35^, rocky intertidal^36^, coral reefs^37^, streams^38^ and intertidal mudflats^24^). Overall, snail grazing caused 50 to 80% reductions in abundance and biomass, and around 10% reductions in diversity and richness. These values are comparable with previous experiments in our study system^39^, but higher than those reported in previous studies conducted on similar systems (i.e., 25–50% reductions^24, 25^).A likely explanation for these stronger grazing impacts could be the extremely high density of snails in our system^25, 39^. Nutrient effects were not as strong in our study; however, these results coincide with similar studies in diatom-dominated systems similar to ours^24, 25^.

All variables directly related to the structure of benthic microalgae showed clear and consistent responses, whereas ecosystem functions showed mixed responses. Given that diatoms (by far, our most abundant group) are well known for their production of EPS^28, 29^, we expected higher EPS concentration in those treatments with higher microalgal abundance. Surprisingly, we found marked impacts of snail grazing on diatoms (data not shown) but no effects on EPS. Orvain *et al*.^40^ found similar results along with high densities of gastropod grazers. They suggested that mucus secretion by the gastropods could increase bound EPS proteins and, thus, buffer the effect of diatoms on EPS. Additionally, we also expected a positive relationship between benthic microalgal abundance and biomass and sediment organic matter content^32^. We found snail grazing reduced sediment organic matter, which coincides with known impacts of herbivores and deposit-feeders in aquatic systems^41, 42^. Sediment water content followed the same pattern as organic matter. This pattern was expected given that these two variables are often correlated in muddy intertidal sediments^33, 34^. In addition, some cyanobacteria (our second most abundant group) commonly use atmospheric N_2_ as a source of N. Thus, we expected that higher diversity and abundance would increase the N_2_-fixation. However, N_2_-fixation is generally low when inorganic N is available^30^. Our results showed the latter pattern with a great reduction (>70%) in N_2_-fixation in fertilized plots. The other key process of the nitrogen cycle is denitrification (i.e., the microbial reduction of nitrate to N_2_). Although this process is not directly affected by benthic microalgae, they can likely affect denitrification indirectly through impacting nitrifying bacteria and the coupled nitrification-denitrification rates^31^. Denitrification requires nitrogen, but also requires organic matter to fuel the process^43^. Coincidentally, we found the highest rates of denitrification on the fertilized exclosures (high nitrate, high organic matter). Interestingly, we also found the lowest rates in unfertilized exclosures. Nitrogen or organic matter limitation is not a likely explanation for these results because unfertilized exclosures did not show reduced nitrate or organic matter concentrations. The most likely explanation is a competitive exclusion of denitrifying bacteria within these plots. This competitive exclusion has been observed between microalgae and ammonia-oxidizing^44^ or nitrifying bacteria^31^).

Ecosystem functions and services may be influenced by different factors, and the idea that trade-offs may emerge if we try to maximize them has been proposed for a decade^2, 7^. Here we evaluated how altered herbivore densities and increased nutrient inputs, two widespread human impacts, affect a variety of functions. Studies evaluating drivers of multiple functions at once are common in terrestrial systems, particularly using biodiversity changes to understand changes in functioning. Indeed, overwhelming evidence shows that biodiversity promotes different ecosystem functions and services^45, 46^. Even more, this relationship increases when several functions are considered simultaneously^9, 47, 48^. Consequently, it was proposed that biodiversity can be as important as other common factors (i.e. fire, nutrients, consumers, carbon dioxide) in driving ecosystem functioning^46^. Surprisingly, these studies, including human impacts and functioning, are comparatively scarce in marine systems. Our study shows that by differentially affecting both structural (i.e. diversity, abundance and biomass of primary producers) and functional aspects of natural systems, human impacts produce decoupled effects between them. In other words, our results reveal that anthropogenic drivers such as higher nutrient inputs and altered consumer populations can have profound impacts on the structure of primary producer’s assemblages, strongly affect a range of ecosystem functions (discussed above), and, nevertheless, do not consistently affect ecosystem functions (i.e. do not lead to an overall altered ecosystem function). We know that the human footprint on Earth affects both biodiversity and ecosystem functioning^1, 6, 49^. We have also been advocating a great effort to understand the biodiversity – multifunctioning relationship, which undoubtedly yielded extremely valuable information. Nevertheless, our knowledge of the relationship between human impacts and multifunctionality is comparatively meager. Our results revealed that changes in functioning were not consistent across functions, and that the sum of all functional changes was mostly neutral. Given that there were clear impacts on most individual functions, these results also emphasize the need to simultaneously evaluate multiple ecosystem functions to avoid misconceptions based on singular results.

## Methods

### Study site

The study was conducted in the Mar Chiquita coastal lagoon (37° 44′ S, 57° 26′ W, Argentina), a UNESCO Man and the Biosphere Reserve. The lagoon is brackish (salinity 0.5 to 34 psu) with low-amplitude tides (≤1 m). The low intertidal zone is characterized by extensive mudflats followed by *Spartina densiflora* and *Sarcocornia* sp.salt marshes at higher elevations^50^. The burrowing crab *Neohelicegranulata* is ubiquitous across the tidal flats and marshes, where it plays a number of important ecological roles^51^. The hydrobiid snail *Heleobia australis*(up to 7 mm in shell length)^52^ is another prominent organism in the lagoon, reaching extremely high densities (>15,000 snails * m^−2^) in low mudflats, and exerting substantial top-down control on benthic microalgal abundance and diversity^39^. We performed our experiments in a 100 × 1 m strip in the low intertidal mudflat along the shoreline.

### Experimental design

In the low mudflat, we carried out a 2 × 3 factorial experiment manipulating nutrients (N and P addition or not) and snail (*H. australis*) density (exclosures, cage controls and controls). The exclosures had very low snail densities and grazing, and the cage controls and controls (open plots) had ambient snail densities and grazing. Cage controls were intended to account for cage effects other than excluding grazers (e.g. decreased water flow) while allowing for ambient grazing. The experiment ran for 13 days in March 2012. Given the extremely high snail densities in the study site^39^, prior to starting the experiment the top 0.5 cm of sediment was scrubbed to remove all snails from the experimental plots (exclosure, cage controls and controls). Additionally, undisturbed control plots were added where snails were not removed. We followed a randomized block design with five blocks and a replicate of each of the seven treatments (non-fertilized exclosure, fertilized exclosure, non-fertilized cage control, fertilized cage control, non-fertilized control, fertilized control, and undisturbed control) randomly located within each block. Each plot was rectangular (25 × 35 cm). Control plots were delineated with plastic stakes in each corner. Plots were separated by 35 cm within blocks, and contiguous blocks were separated by at least 1 m.

Exclosures were built with bottomless transparent rectangular plastic containers 15 cm in height. The bottomless edge of the container was buried 3 cm into the sediment, and the lid secured to the top of the container. To enhance air and water flow through the exclosures, large openings covering most of the exclosure surface were cut into all four sides and covered with 1- mm mesh that prevented snail entrance. To maximize light penetration, most of the lid surface was replaced with a transparent PVC film (150 μm) that also had a 7 × 9 cm mesh (1 mm) window in the center to allow for air and water flow through the top of the exclosure (Supplementary Fig. 1). Cage controls were built to the same specifications as the exclosures, but with an open strip cut 1-cm high from the sediment along the base of each side to allow for snail entrance (Supplementary Fig. 1).

Nitrogen (1.5 g of pelletized urea per fertilized plot and day of fertilization) and P (0.5 g of pelletized sodium phosphate per fertilized plot and day of fertilization) were added at the beginning of the experiment, at day 4, and at day 8. These values were based on realistic anthropogenic nutrient loads measured for other ecosystems^53, 54^ and amended to reach a 16N:1P fertilization ratio (i.e. ratio for optimal growth rates for benthic microalgae)^55^. We bagged the nutrient pellets in 2 × 4 cm fine-mesh nylon cloth bags. Four bags, each containing a quarter of the quantities described above, were carefully buried 5 cm into the sediment equidistantly throughout the plot, and their location marked with wire stakes. On each fertilization day, all old bags were carefully removed and new ones deployed at the same location. There were no differences in baseline nutrient concentrations among treatments (Supplementary Table 2), while fertilization effectively increased nutrient concentrations (Supplementary Table 3). Almost daily we counted and removed all the snails present within the exclosures. On average, we found 5 snails per exclosure, except for the first visit when we found and removed ~113 snails per exclosure. Our exclosures were successful at reducing snail density (Supplementary Table 4). The plastic and mesh on the exclosures and cage controls was carefully and mechanically cleaned during each visit to remove fouling algae and sediment. Exclosures only reduced incident PAR on the sediment at noon by 5% compared to ambient conditions. However, they reduced water flow (estimated using dissolution rates of chlorine pills on an additional subset of plots) by about 25% in relation to ambient conditions. This effect was partially captured by the cage controls, but not fully, since exclosures still showed a 9% reduction in flow in relation to cage controls (Supplementary Table 5). Additionally, the comparison of disturbed vs undisturbed control plots revealed that only the variables related to the community structure of benthic microalgae were affected by the pre-treatment sediment removal (Supplementary Table 6).

### Variables measured

We measured the concentrations of dissolved inorganic nitrogen (DIN = nitrite + nitrate + ammonium) and PO_4_^3−^ in the sediment porewater on day 0 before fertilizing the sediment, and on days 3, 6, 9 and 13. These measurements were taken in a subset of the plots (i.e. non-fertilized and fertilized exclosure and control plots), as well as undisturbed controls, in the same three blocks. On the last day of the experiment we measured (7 plots per block x 5 blocks = 35 plots) the abundance (identified to genera)^39^ and biovolume^56^ of benthic microalgae, and a number of metrics indicative of ecosystem properties or functionality (i.e., N_2_-fixation, denitrification, extracellular polymeric substances (EPS), sediment organic matter and water content) in all plots. As in other studies, we considered ecosystem functions to responses (including fluxes and standing stocks) that were relevant to the functioning of the ecosystem^9^. Although several studies use biomass as a proxy for productivity in multifunctionality studies^9, 27, 46^, we did not include biovolume (as a proxy for biomass) as another ecosystem function. These two variables (biomass vs primary production) have different meanings and implications, as primary production corresponds to the amount of CO_2_ fixed through photosynthesis into new microalgal biomass, whereas biovolume (biomass) is an indicator of the production left-over that is not lost through processes such as grazing (which we showed to be high in our study), export and burial (i.e. remaining biomass after accounting for production losses).

Porewater nutrient samples were collected with a 5 ml syringe inserted 5 cm deep into the sediment and concentrations measured using spectrophotometric methods^57, 58^. All snails present in a 10 × 10 cm quadrat tossed randomly within each plot were counted. A 2 × 1 cm (diameter x depth) core was extracted for the benthic microalgal analysis^56^. N_2_-fixation and denitrification were measured using separate *in situ* incubations (90 minutes in 10 cm diameter, 300 cm^3^ chambers) following the isotope pairing technique for denitrification (using 50 μM ^15^N-NO_3_^−^)^59^ and the acetylene reduction assay for N_2_-fixation^60^. Samples were collected by syringe, at the beginning and at the end of the incubation period following Fanjul *et al*.^61^. Linearity was checked in previous works at this same study site. Our N_2_-fixation rates from unfertilized plots were similar to those previously observed in a tidal flat nearby^61^. One 2 × 1 cm core per sample was extracted to quantify EPS^62^. Organic matter and water content were estimated by collecting one 3 × 3 cm core per plot. These samples were weighed, oven-dried and weighed again to estimate water content. Then, a known dry weight subsample was burned (550 °C, 6 hours) to calculate the ash-free dry weight to estimate organic matter content. All samples were collected haphazardly within each plot, with no overlap of sampling/incubation locations.

### Structural and functional changes

We used diversity (i.e. richness: number of genera, the lowest taxonomic level arrived at in our samples), abundance and biomass (estimated as biovolume ref. 56) as measures of community structure. The raw data used in this section is the same described in the previous section. The effects of top-down and bottom-up forces on these community structure indices, proxies for ecosystem functions, and nutrient concentrations were evaluated with linear mixed-effects models (considering block as the random term). Visual inspection of residual plots did not reveal any obvious deviations from homoscedasticity or normality. The final model was selected using the likelihood ratio test following Zuur *et al*.^63^ by using the *lmer* function from the lme4 package^64^ for R 3.2. Top-down effects were evaluated comparing cage controls and exclosures. These two treatments did not exhibit differences in PAR interception, pelagic microalgae abundance (~5%), and relatively minor differences in water flow. Nevertheless, we also provided the results of control plots for each variable in order to ease the comparison of the results with “uncaged” treatments. To evaluate differences between disturbed and undisturbed controls, these two treatments were compared for each dependent variable using the same tests described above.

### Consistency in structural and functional changes

To evaluate if different structural or functional variables followed similar patterns across treatments, we standardized them to remove differences in measurement scale. We used the z-transformation because it is commonly used and useful for traditional linear statistics^47^. Given that different functions may not respond similarly to a given treatment^8^, we also evaluated the overall functional and structural changes. Here we used the difference in mean standardized structural or functional variables between treatments to estimate the overall structural or functional change across treatments. This index can be expressed as (C_*f*_ − NC_*f*_), where *C*_*f*_ is the observed value of the treatment including a given factor (i.e. nutrient addition or presence of herbivores) for structure or function *f*, *NC*_*f*_ is the observed value of the treatment without including that factor (i.e. ambient nutrients or absence of herbivores) for structure or function *f*. We calculated one index per structure or function for treatments manipulating nutrients (C = addition − NC = ambient nutrients) for each of both herbivore treatments, and one index per structure or function for treatments manipulating herbivores (C = cage control and NC = exclosure) for each nutrient treatment. We then performed a one-sample t-test for each of the four groups to evaluate if any of the groups significantly deviated from 0.

## Electronic supplementary material

Supplementary Figures and Tables
