# Supplementary material for: Nutrient and herbivore alterations cause uncoupled changes in producer diversity, biomass and ecosystem function, but not in overall multifunctionality
Source: Sci Rep. 2017 Jun 1;7:2639. doi: 10.1038/s41598-017-02764-3 (PMC5454014; doi:10.1038/s41598-017-02764-3)
Supplement: Supplementary file 1 — Supplementary Figures and Tables [file 41598_2017_2764_MOESM1_ESM.pdf]

Nutrient and herbivore alterations cause uncoupled changes in producer diversity, biomass and ecosystem function, but not in overall multifunctionality

Alberti, J.[1]*; Cebrian, J.[2]; Alvarez, F.[3] ; Escapa, M[1].; Esquius, K.S.[4], Fanjul, E.[1]; Sparks, E.L.[5]; Mortazavi, B.[2]; Iribarne, O.[1]

[1] Instituto de Investigaciones Marinas y Costeras (IIMyC), UNMdP – CONICET, Mar del Plata, Argentina

[2] Dauphin Island Sea Lab, Dauphin Island, AL 36528 - University of South Alabama Department of Marine Sciences, Mobile, AL 36688, US

[3] Laboratorio Cuenca del Salado, Instituto de Limnología "Dr. Raúl A. Ringuelet" (ILPLA) (CONICET – UNLP), La Plata, Argentina

[4] Laboratorio de Limnología, Departamento de Biología, Universidad Nacional de Mar del Plata, Mar del Plata, Argentina

[5] Mississippi State University Coastal Research and Extension, Biloxi, MS 39532 – Mississippi-Alabama Sea Grant Consortium, Ocean Springs, MS 39564, US

* Corresponding author: jalberti@mdp.edu.ar

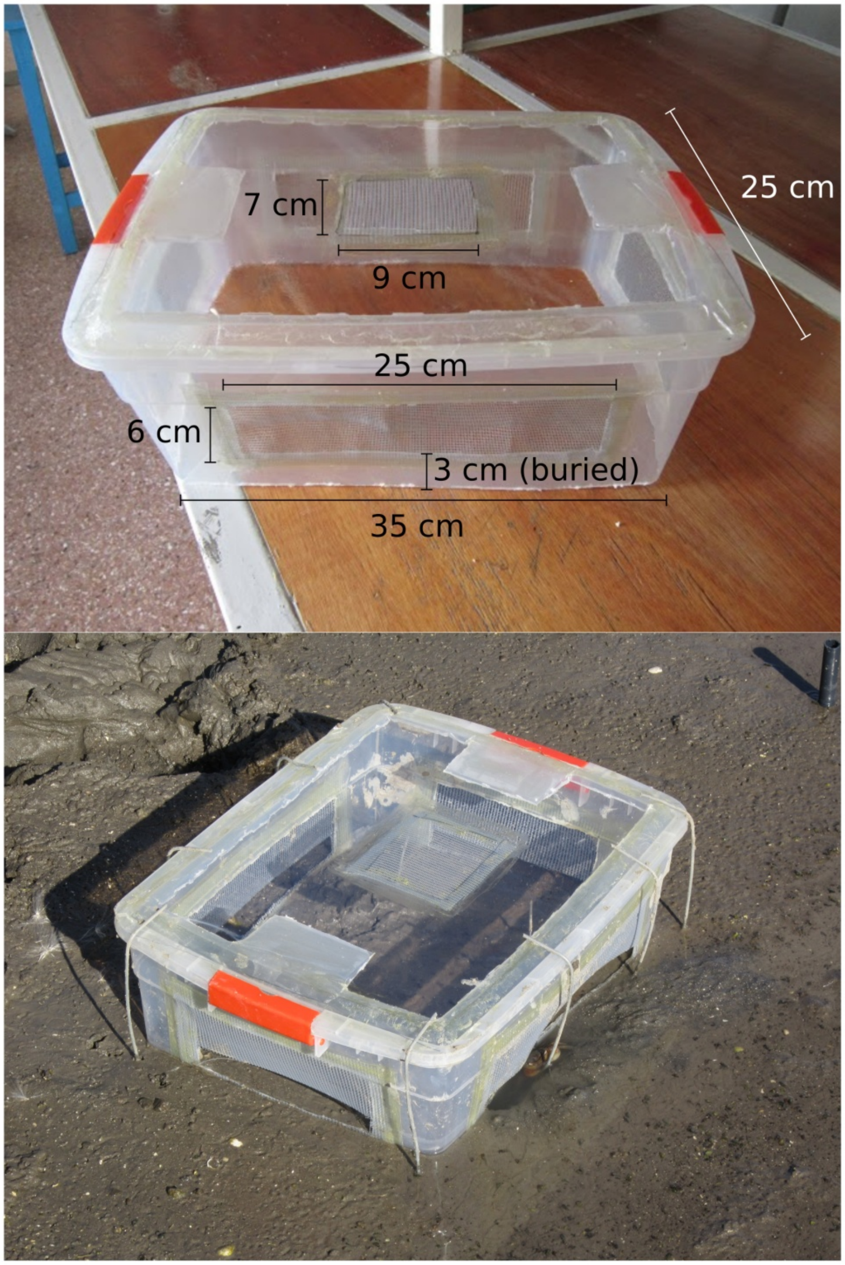

Fig. S1

Shape and dimensions of cage controls and exclosures. The top figure shows the dimensions of an exclosure as well as the dimensions of the plastic mesh sizes to allow water flow. The bottom figure shows a cage control settled in the field. Notice the openings in the plastic mesh to allow free movement of snails can be seen at the ground level.

Table S1

Results of the linear mixed models for structural and functional variables, taking nutrients and snail grazing as fixed factors and block as the random effect. Significance and model selection was performed following Zuur et al. [63]. Statistics in italics denote that the F statistic was used instead of the χ^2 because the random effect term was not significant.

Significant term	χ^2 - *F*	df	p	Grazing treatment	p (with vs without nutrients)	Comparison of grazing treatments	p (ambient)	p (fertilized)
Abundance of benthic microalgae (log-transformed)								
Grazing * nutrients	29.16	2	< 0.001	Exclosure	0.995	Exclosure vs cage control	< 0.001	< 0.001
				Cage control	0.997	Exclosure vs grazed	< 0.001	< 0.001
				Grazed	< 0.001	Cage control vs grazed	< 0.001	< 0.001
Diversity								
Grazing * nutrients	12.55	2	0.002	Exclosure	0.16	Exclosure vs cage control	0.94	0.002
				Cage control	0.66	Exclosure vs grazed	< 0.001	0.02
				Grazed	< 0.001	Cage control vs grazed	< 0.001	0.82
Biovolume (log-transformed)								
Grazing	*101*	2	< 0.001			Exclosure vs cage control	< 0.001	
						Exclosure vs grazed	< 0.001	
						Cage control vs grazed	0.35	
Sediment organic matter (log-transformed)								
Grazing	14.20	2	< 0.001			Exclosure vs cage control	< 0.001	
						Exclosure vs grazed	0.02	
						Cage control vs grazed	0.23	
Sediment water content (log-transformed)								
Grazing	17.19	2	< 0.001			Exclosure vs cage control	< 0.001	
						Exclosure vs grazed	< 0.001	
						Cage control vs grazed	0.68	
EPS								
Grazing * nutrients	9.07	2	0.011	Exclosure	0.81	Exclosure vs cage control	0.99	0.76
				Cage control	0.038	Exclosure vs grazed	0.83	0.53
				Grazed	0.55	Cage control vs grazed	0.62	0.028
N_2-fixation (log-transformed)								
Nutrients	*21.7*	1	< 0.001					
Denitrification (log-transformed)								
Grazing * nutrients	*15.4*	2	< 0.001	Exclosure	< 0.001	Exclosure vs cage control	0.014	0.035
				Cage control	0.96	Exclosure vs grazed	0.003	0.198
				Grazed	0.89	Cage control vs grazed	0.54	0.95

Table S2

Baseline (right before starting the experiment) dissolved inorganic nitrogen (DIN) concentrations (μM) in the different treatments. Phosphate (PO_4^{-3}) concentrations were below the detection threshold in all treatments and were not analyzed. Results of the linear mixed model, taking nutrients and snail grazing as fixed factors, and blocks as the random effect. Significance and model selection was performed following Zuur et al. [63]. Here and in the next Tables we only report non-significant terms when this information is relevant. These results show that there were no baseline differences in nutrient concentration between the different treatments.

Treatment	F	df	p	Mean DIN (μM) concentration (SE)			
				Ambient	Fertilized	Exclosure	Grazed
Nutrients	0.19	1	0.67	28.28 (7.41)	31.30 (6.35)		
Grazing	1.65	1	0.23			25.65 (6.40)	33.93 (6.50)

Table S3

Dissolved inorganic nitrogen (DIN) and phosphate (PO_4^{-3}) concentrations in the different treatments along the experiment. Results of the linear mixed model, taking nutrients, snail grazing and sampling date as fixed factors, and plots nested in blocks as the random effect. Significance and model selection was performed following Zuur et al. [63]. These results show that fertilization effectively increased nutrient concentrations (32.8 % for DIN and 23.7 % for PO_4^{-3}).

Significant term	X^2	df	p	Mean DIN and PO_4^{-3} concentrations (SE)			
				Ambient	Fertilized	Exclosure	Grazed
DIN (μM)							
Nutrients	7.90	1	0.005	31.31 (6.21)	41.59 (11.29)		
Grazing	7.42	1	0.006			31.51 (6.25)	41.39 (11.33)
Day	20.36	3	< 0.001				
PO_4^{-3} (μM)							
Nutrients	4.63	1	0.031	2.70 (0.40)	3.34 (0.65)		

Table S4

Snail counts (i.e. individuals per 100 cm^{-2}) in the different treatments at the end of the experiment. Results of the linear mixed model, taking nutrients and caging treatment (exclosure, cage control or control; factor previously named as snail grazing) as fixed factors and blocks as the random effect. Significance and model selection was performed following Zuur et al. [63]. Different letters (between brackets) denote significant differences between those treatments. These results show that exclosures strongly reduced snail densities in the experimental plots, and that our fertilization did not affect snail densities.

Treatment	F	df	p	Mean snail counts (SE)				
				Exclosure (a)	Cage control (b)	Grazed (b)	Ambient	Fertilized
Caging	251.7	2	< 0.001	3.8 (1.7)	247.3 (40.5)	280.3 (35.0)		
Nutrients	0.06	1	0.81				192.4 (86.1)	161.9 (79.6)

Table S5

Effects of treatments manipulating snail grazing on water flow. It was estimated using chlorine pills on five exclosures, cage controls, and controls once the experiment was finished. After a week, chlorine pills were collected, dried and weighed, and the percent weight lost was calculated. Linear mixed models were used to evaluate the potential undesired effects of caging, taking nutrients and caging treatment (exclosure, cage control or control) as fixed factors and blocks as the random effect. The full model in R-language notation was:

lmer(water_flow ~ caging_treatment + (1 | block), data = data)

where water_flow was chlorine pill percent weight lost. Significance and model selection was performed following Zuur et al. [63]. Different letters (between brackets) denote significant differences between those treatments. These results show that exclosures and cage controls reduced water flow by ~ 32 %. Although water flow reduction was higher in exclosures (i.e. less % weight lost) than in cage controls, this was only a 5.8 % difference.

	χ^2	df	p	Exclosure	Cage control	Grazed
Water flow	37.44	2	< 0.001	28.5 (0.68) (a)	34.3 (1.44) (b)	45.8 (1.06) (c)

Table S6

Comparison of disturbed versus undisturbed control plots (using paired t-tests) to detect potential undesired effects after removing the top layer of sediment to exclude snails from experimental plots. We performed this analysis for all the community structure variables (abundance of benthic microalgae, biovolume and diversity), all the proxies for ecosystem functions (N_2-fixation, denitrification, EPS, sediment organic matter and water content) that we measured, and some environmental variables (snail density, DIN and PO_4^{-3} concentrations). These results show that only the variables related to the community structure of benthic microalgae were affected by the pre-treatment sediment removal.

Dependent variable	Mean (SE)		t	p
	Undisturbed control	Disturbed control		
Abundance of benthic microalgae	208.45 (93.22)	488.67 (218.54)	2.971	0.041
Biovolume	0.23 (0.10)	0.38 (0.17)	2.405	0.070
Diversity	13.0 (5.81)	17.4 (7.78)	3.317	0.030
N_2-fixation	35.63 (15.94)	36.20 (16.19)	0.054	0.960
Denitrification	4.28 (1.92)	3.97 (1.78)	-0.654	0.549
EPS	28.62 (12.80)	29.21 (13.06)	0.070	0.948
Sediment organic matter	3.23 (1.44)	3.28 (1.47)	0.092	0.931
Water content	42.39 (18.96)	39.40 (17.62)	-0.964	0.390
Snail density	267.6 (119.67)	327.6 (146.51)	1.804	0.145
DIN	32.40 (14.49)	22.70 (10.15)	-0.70	0.523
PO_4^{-3}	4.15 (1.86)	2.76 (1.23)	- 1.09	0.338
